# Structures and Metabolic Properties of Bovine Milk Oligosaccharides and Their Potential in the Development of Novel Therapeutics

**DOI:** 10.3389/fnut.2019.00050

**Published:** 2019-04-24

**Authors:** Randall C. Robinson

**Affiliations:** Department of Food Science and Technology, University of California, Davis, Davis, CA, United States

**Keywords:** milk, prebiotic, obesity, mass spectrometry, therapeutic

## Abstract

Among the many bioactive components in human milk, the free oligosaccharides (OS) have been intensely studied in recent decades due to their unique ability to selectively modulate the infant gut microbiota, in addition to providing numerous other health benefits. In light of the demonstrated value of these compounds, recent studies have set out to characterize the structures and properties of the similar and more widely-available OS in the dairy industry. This mini review gives a brief overview of the common analytical techniques used to characterize bovine milk OS and highlights several recent, key studies that have identified valuable physiological and metabolic effects of these molecules *in vivo*. Although traditionally considered indigestible by human enzymes, evidence now suggests that milk OS are partially absorbed in the intestines and likely contribute to the development of molecular structures in the brain. Furthermore, aside from their prebiotic effects, these compounds show promise as therapeutics that could alleviate numerous metabolic abnormalities, including undernutrition, obesity, and excessive intestinal permeability. The need for novel treatments to address these and related health issues is motivating the development of scalable techniques to produce large quantities of milk OS for use as food ingredients. The safety and tolerability of high dosages of bovine milk OS have been demonstrated in two independent human studies, which potentially opens the door for further research aiming to utilize these molecules to alleviate common metabolic health issues.

## Introduction

Milk harbors a suite of bioactive compounds, including free oligosaccharide (OS) structures that are well-characterized as selective prebiotics and that play an important role in infant health and development (1, 2). Research in the last few decades has made immense progress in characterizing the beneficial biological functions of OS and uncovering the mechanistic pathways by which they are exerted. The majority of studies on milk OS functionality initially focused on the highly-concentrated OS in human milk. Human milk OS have been extensively profiled, with as many as 200 structures being identified in comprehensive studies (3, 4), and the ability of gut-associated bacteria to consume many of these structures is well-documented (5–7). Several gut bacterial species, including select species of *Lactobacillus* and *Bifidobacteria*, are highly desirable due to their ability to down-regulate an over-active immune system and reduce inflammatory response (8). This research field has now expanded to identify the similar structures and bioactivities of milk OS from other mammalian species. In particular, industrial production of bovine milk has prompted studies into the therapeutic value of bovine milk oligosaccharides (BMOs) and the dairy industry's relatively underutilized BMO-containing side streams. This mini-review highlights recent studies that demonstrate novel bioactivities of BMOs, with a particular focus on their digestibility and metabolic effects. The paper also provides an overview of the current analytical tools used in OS characterization and the development of industrial-scale processes for BMO production. Considering the wide availability of BMOs in dairy streams and the current need for therapeutics with BMO-like functionalities, these molecules show promise as a solution to epidemic metabolic and digestive illnesses.

## Bovine Milk Oligosaccharide Composition and Structures

**Figure 1:**
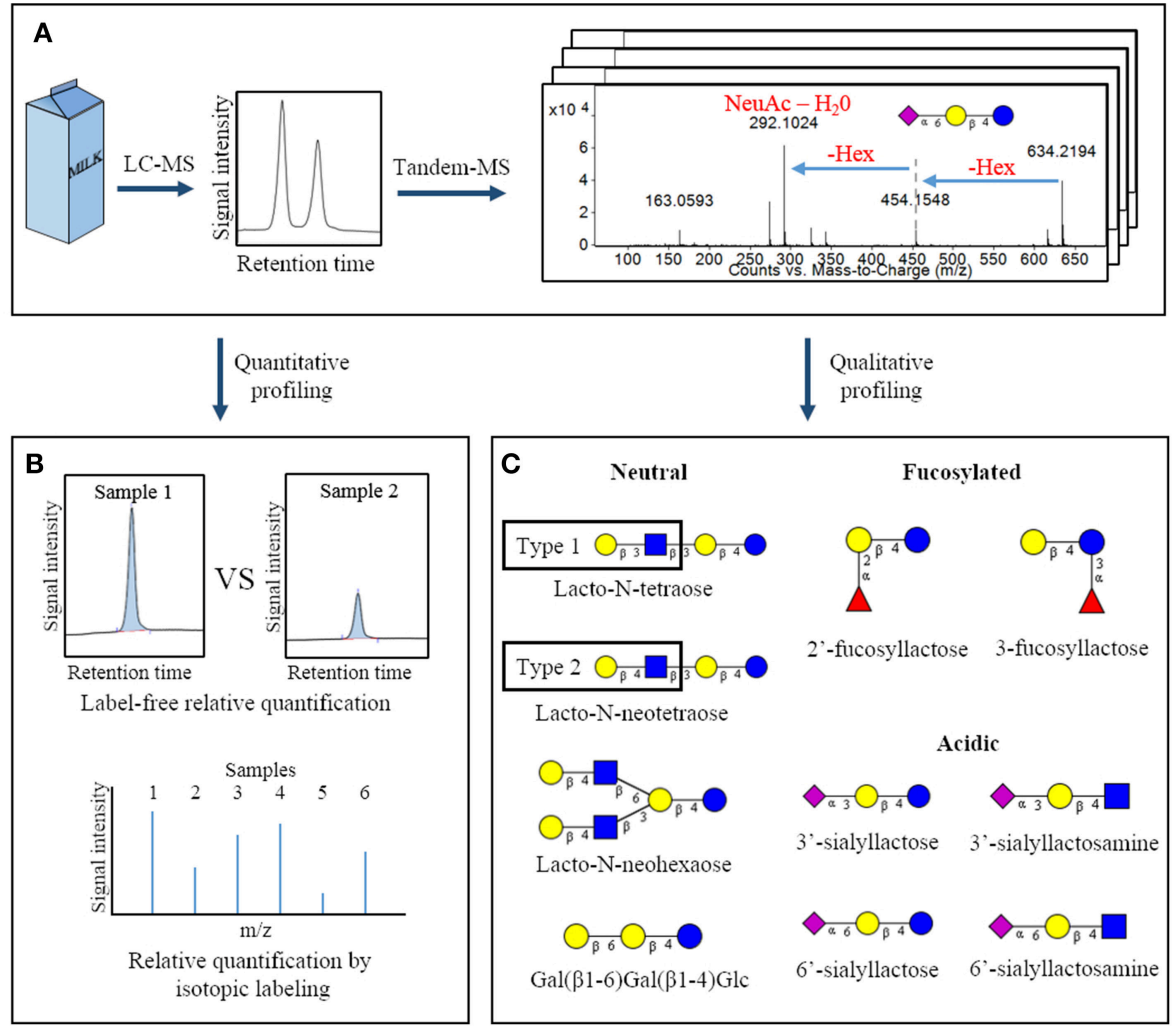
Overview of common analytical methodologies used to study milk oligosaccharides. Following a suitable extraction procedure, bovine milk is often analyzed by liquid chromatography-mass spectrometry to identify the oligosaccharides present in a sample and measure their abundances **(A)**. Quantification can be conducted using peak heights or areas (label-free quantification) or by isotopic labeling strategies **(B)**. Through analysis of tandem-MS data and comparison with analytical standards, specific oligosaccharide structures present in a sample can be deduced. This panel represents a selection of oligosaccharides with fully-elucidated structures that are found in both bovine and human milk **(C)** (11). Hex, hexose; NeuAc, N-acetylneuraminic acid. 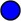 – glucose; 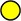 – galactose; 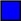 – N-acetylglucosamine; 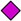 – N-acetylneuraminic acid; 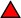 – fucose.

Oligosaccharides in bovine milk are assembled in the mammary gland by combining the monosaccharides glucose (Glc), galactose (Gal), N-acetylglucosamine (GlcNAc), N-acetylgalactosamine, fucose, and the sialic acids N-acetylneuraminic acid and N-glycolylneuraminic acid (9, 10). The OS structures contain either lactose (Gal(β1-4)Glc) or N-acetyllactosamine (Gal(β1-4)GlcNAc) at their reducing end, with additional monosaccharide residues branching off from the non-reducing galactose (9, 10). In some cases, the BMOs possess lacto-N-biose (Gal(β1-3)GlcNAc) or N-acetyllactosamine units linked to the lactose core, which are defining features of the type 1 and type 2 OS structures contained within many human milk OS (Figure 1C) (1, 9). The collection of BMOs found in milk and colostrum has been extensively profiled by our research group, with 30–50 structures typically being identified in comprehensive studies (9, 12–14). Although bovine milk contains fewer OS structures than human milk, the two share at least 10 common structures (Figure 1C), including the acidic 3′-sialyllactose and 6′-sialyllactose. These two OS comprise a large percentage of the BMO pool (9, 11). Complete or partial structures are known for many BMOs (9); however, there remains a large proportion of BMOs for which only monosaccharide compositions are known. For example, recent studies have identified several large fucosylated OS in bovine milk, but to date only the monosaccharide compositions have been determined (9, 15, 16). A more complete structural characterization of the entire BMO pool could improve our understanding of their bioactivities and digestibilities by microbes. In many cases, linkage types influence functionality, as exemplified by *in vivo* studies showing the ability of α1-2–linked fucosyloligosaccharides to prevent *Campylobacter jejuni* infection (17).

## Analytical Methodologies for Milk Os

Identification of the individual OS structures in milk has come with numerous analytical challenges, many of which have been resolved in the past two decades. The free OS in milk are frequently analyzed by liquid chromatography-mass spectrometry (LC-MS, Figure 1A) (12, 16, 18). This technique is utilized for initial discovery and profiling of the entire collection of OS in a sample. It conveniently provides relative compound abundances and monosaccharide compositions for a multitude of OS within a single experiment. Common LC-MS strategies for OS analysis have been recently reviewed in greater detail elsewhere (19). However, the potential for branching within an OS structure, as well as the numerous possible linkages between neighboring monosaccharides, often requires further experiments to achieve complete structural elucidation.

In some cases complementary techniques are used along with MS for in-depth characterization of OS structures. For example, Aldredge et al. (9) fractionated the bovine milk OS pool by high performance liquid chromatography, incubated each bovine milk OS with glycosidases of known specificity, and analyzed the changes produced with LC-MS. This labor-intensive approach determined a variety of glycosidic linkages and specific monosaccharide types for numerous OS (9). A similar approach was used by Wu et al. in the determination of human milk OS structures (3, 20). Alternatively, more rapid approaches that do not rely on pre-fractionation use strategic derivatization and subsequent analysis of the hydrolyzed monosaccharides to provide at least partial linkage information. Galermo et al. have recently published a method by which the monosaccharides and linkage types present in oligo- and polysaccharides can be determined in a high-throughput manner using a pair of derivatization strategies and LC-MS (21).

Not surprisingly, the multitude of potential glycosidic linkages and the efforts required to deduce complete OS structures has hindered chemical synthesis of larger OS structures for use as analytical standards. However, several standards are now available for the smaller bovine milk OS, allowing absolute quantification of a subset of the OS ensemble (18, 22). When analytical standards are unavailable, OS abundances are often measured in relative terms. This can be done using measures such as mass spectral peak height or chromatographic peak area of OS to compare abundances among samples, known as a “label free” relative quantification. Alternatively, several derivatization strategies have been developed that allow relative abundances of isotopically-labeled carbohydrates to be compared on the basis of the intensity of their unique mass spectral peaks. Some examples include reducing-end derivatization of glycan sample pairs with a heavy/light label pair (23, 24) or with a series of isobaric reagents (Figure 1B) (25, 26). Further details of these relative quantification techniques and derivatization strategies have been described in a recent book chapter by Orlando (27) and in a review by Dong et al. (28), Robinson (29).

## Bovine Milk Oligosaccharide Content and Known Phenotypic Variations

**Figure 2:**
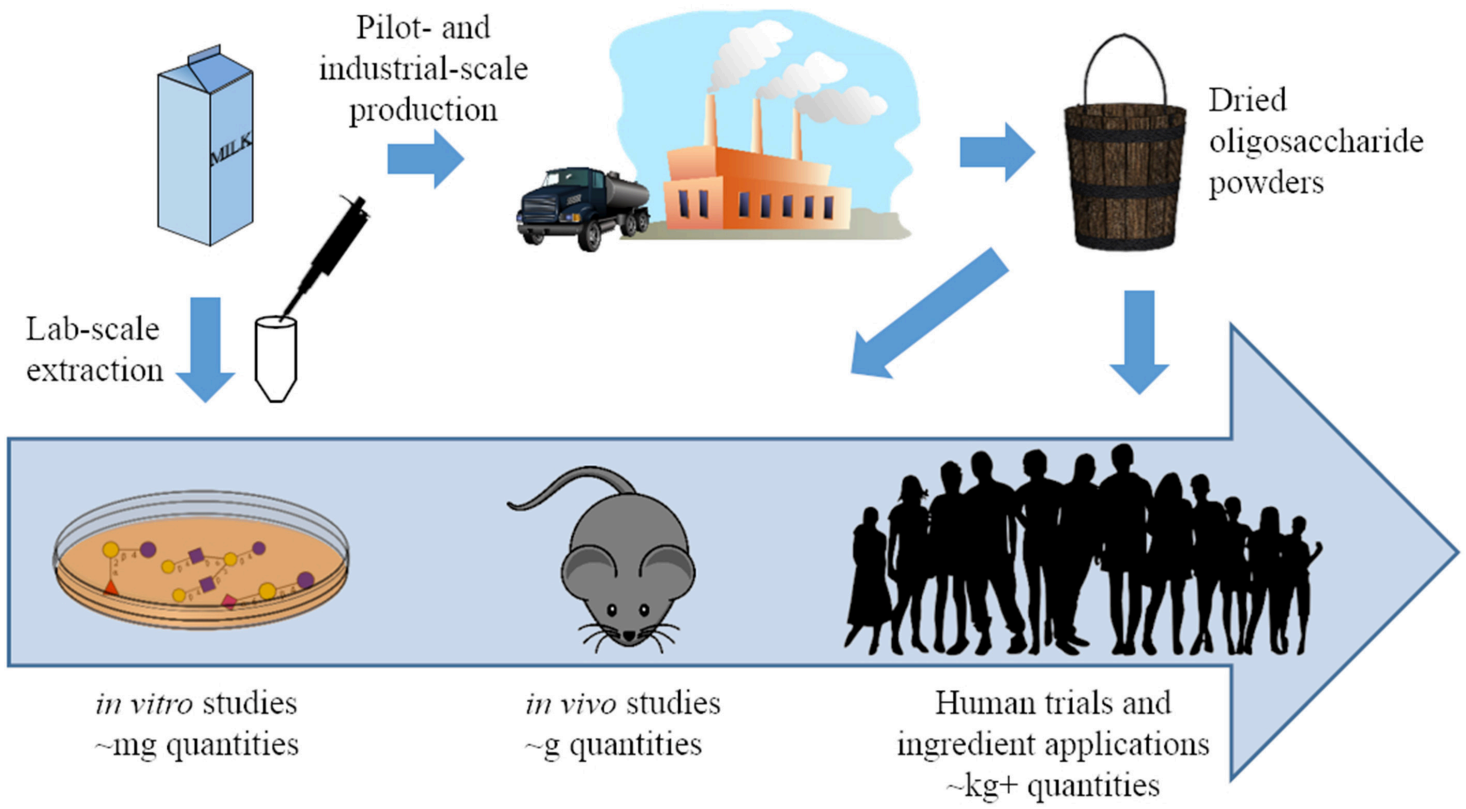
A pictorial representation of the progress made in milk oligosaccharide functional testing and isolation strategies over the past 20 years. Previously, functional testing was generally limited to *in vitro* studies conducted with oligosaccharides isolated from milk at lab scale. Synthetic and pilot-scale isolation can now generate these compounds in larger quantities, and oligosaccharides from both sources have been used in *in vivo* studies and clinical trials (37).

Although analytical standards for quantification do not yet exist for many BMOs, the total OS concentration in bovine milk is estimated at approximately 1–2 g/L in colostrum and 100 mg/L in mature milk (18, 30, 31). Despite the current difficulties in quantifying some OS structures, analytical studies have deduced a wealth of relevant information on OS production in cows. During the first week of lactation, BMO abundances drop relatively quickly and decline somewhat further as cows transition to mature milk (14, 18). Bovine colostrum is a particularly rich source of these OS, and processing streams within the dairy industry have the potential to serve as a raw material for BMO isolation. During cheese production, non-casein proteins and polar molecules such as salts, lactose, and BMOs are eliminated from the cheese as whey. Purification of the whey proteins produces a liquid byproduct known as whey permeate, a stream that contains BMOs (32). Although uses for whey permeate have been identified, it is often considered a waste stream (33), and dried whey permeate and its byproducts are typically sold at low prices (34). Therefore, recovery of BMOs from whey permeate could add value to this stream and improve dairy industry sustainability. Pilot-scale techniques to isolate OS from this dairy stream have been developed using membrane filtration (35, 36). The wide availability of dairy side streams that contain BMOs could allow these processing techniques to feasibly produce isolated milk OS for functional testing and therapeutic applications (Figure 2). As described in the following sections, isolated BMOs have demonstrated beneficial health effects in a variety of *in vivo* studies.

Several studies have explored variations in milk OS abundances within dairy cattle in order to characterize industrial BMO availability and to elucidate factors that influence OS production. The Holstein-Friesian and Jersey breeds are commonly used for milk production, and several studies have examined differences in OS production among these and related breeds (14, 16, 30). Most recently, we have profiled milk OS abundances in a total of 634 samples collected from these breeds and have measured greater amounts of most OS in milk from the Jersey breed (38). The Jersey cows, however, also showed much greater cow-to-cow variability. In light of the fact that environmental sources of variation were controlled, these results may reflect an underlying genetic influence on OS production. A recent study by Liu et al. examined the OS content of genotyped Australian Holstein cows and measured high heritabilities for many OS, indicating that the variation in OS abundances between cows were substantially influenced by genetics (39). The study also identified numerous quantitative trait loci, or regions of the genome which likely influence OS abundances (39). Further studies on this topic will be integral to complete elucidation of the pathways responsible for BMO synthesis. While it is suspected that free milk OS are synthesized by some of the same enzymatic pathways that are used in protein-linked glycan synthesis, investigating the genetic influence on OS production should provide more concrete proof of this possibility. This knowledge could also enable implementation of selective breeding strategies to increase the levels of BMOs in milk without requiring genetically modified organism-based approaches.

## Digestibility: Localized and Systemic Activities

Free milk OS are typically considered indigestible by human enzymes (40–42). Nonetheless, there are several reports of human milk OS existing in infant blood (43) and urine (44–46), indicating that a portion of these molecules are absorbed and circulate in the body. The degree of absorption appears to vary substantially by structure (44), and the biological implications of this absorption have yet to be fully elucidated. It has been hypothesized that absorbed OS can prevent urinary tract infections in infants (47), and recent *in vivo* evidence demonstrates that consumption of 3′-sialyllactose and 6′-sialyllactose increases brain ganglioside-bound sialic acid content in piglets (48). Dietary supplementation with various forms of sialic acid (free or bound to milk OS or protein-linked glycans) has improved learning and increased brain sialic acid content in animal studies (49–51), suggesting that these carbohydrates make an important contribution to brain development.

Milk OS that are not absorbed are available for consumption by the gut microbiota. Human milk has long been known to influence the development of the infant gut microbiota in ways that confer health benefits to the infant, and more recent studies have determined that the milk OS are key to providing this prebiotic functionality (1, 5, 7, 11, 52). These OS selectively feed specific bacterial species that possess the enzymes necessary to metabolize the wide variety of glycosidic linkages found in OS (7, 11, 53). Several of these prebiotic OS from human milk, including lacto-N-tetraose and the sialyllactose isomer pair, are also found in bovine milk (9, 40), and the ability of the BMO ensemble to modulate the gut microbiota *in vivo* has recently been demonstrated (54, 55). Considering the wide availability of dairy side streams from which these OS can be isolated, BMOs show promise as future therapeutics that could be used to provide human milk OS-associated health benefits to infants and adults at a large scale. Initial studies utilizing these OS from dairy streams have revealed a variety of metabolic benefits resulting from BMO consumption, which are reviewed in the following section.

## Metabolic Effects

A well-studied metabolic impact of prebiotic carbohydrates is the ability to indirectly influence short-chain fatty acid (SCFA) production in the intestine by promoting the growth of SCFA-generating bacteria (56, 57). SCFAs are products of anaerobic bacterial fermentation that occurs in the gastrointestinal tract. The major SCFAs produced by the gut microbiota are acetate, butyrate, and propionate. These bacterial metabolites are used as substrates for a variety of host processes, including cholesterol synthesis and gluconeogenesis in the liver, as well as serving as a key energy source for colonocytes (58). The bacterial genera *Bifidobacterium* and *Bacteroides*, each of which contain well-characterized milk OS consumers (5, 7, 59), are contributors to SCFA production (56, 60). Recently, our research group has shown that BMO supplementation alone significantly increased the expression of butyrate-generating bacterial genes in western diet-fed mouse models (55). Aside from being the preferred energy source for colonocytes (58), butyrate can have anti-inflammatory effects in the liver and colon (61).

Isolation of milk OS from dairy streams has enabled experiments identifying novel metabolic effects of OS *in vivo*. A study by Charbonneau et al. used animal models of infant undernutrition to show that dietary supplementation with BMOs provides a microbially-mediated increase in lean body mass and bone growth, and generates metabolite profiles indicative of improved nutrient utilization (62). These results were characterized in both gnotobiotic mice and piglets, and they provide striking evidence that milk OS, in combination with the gut microbiota, play a substantial role in development and regulation of metabolic pathways. Although other non-milk carbohydrate polymers, such as inulin, share some of the properties of milk OS, this study revealed that the metabolic changes induced by milk OS were not duplicated with inulin supplementation (62). Therefore, the unique functionalities of milk OS may be imparted by their higher diversity of monosaccharide types and linkages compared to the less structurally diverse prebiotic polymers.

The availability of a pilot-scale supply of milk OS has also led to key experiments demonstrating the beneficial effects of OS on the development of obesity and intestinal permeability. With the prevalence of overweight adults reaching nearly 40% worldwide, and obesity at 13% worldwide (63), novel strategies to combat this unfavorable metabolic state could lead to widespread improvements in health status and reduce healthcare costs arising from obesity-associated illnesses. A growing body of evidence is establishing a causal relationship between gut microbial dysbiosis, intestinal permeability, and the onset of obesity. Weight gain from diet-induced obesity occurs simultaneously with altered intestinal permeability and gut microbial profiles, as well as decreases in anti-inflammatory cytokine expression (64, 65). Furthermore, transplantation of the gut microbiota from an obese individual to germ-free mice can induce elevated weight gain in the mice (66, 67), highlighting the gut environment as a potential target for therapeutic interventions. Though not entirely understood, it is possible that obesity onset is at least partially initiated by increases in circulating bacterial endotoxin as a result of altered intestinal permeability, as outlined in a previous review (68). Therefore, treatments that maintain gut barrier function and/or reduce endotoxin circulation could lead to viable interventions to prevent obesity and its related metabolic conditions. Studies investigating the ability of BMOs to modulate intestinal permeability have shown promising results. Hamilton et al. recently showed that consumption of an ensemble of BMOs can significantly reduce weight gain and reduce the intestinal permeability that is induced in mice consuming a high-fat diet (54). Dietary supplementation with BMOs also increased SCFA abundance in the cecum, an effect that was not replicated by inulin supplementation (54). In a similar study, introduction of BMOs to the diet of high fat-fed mice, in combination with a weekly gavage of the probiotic *Bifidobacterium longum* subspecies *infantis*, prevented increases in intestinal permeability otherwise associated with the high-fat diet (69).

The prebiotic effect of milk OS could be another significant factor contributing to obesity prevention. The presence of bifidobacteria in the mouse gastrointestinal tract is correlated with reduced plasma and intestinal endotoxin levels (70, 71). Conversely, the gut microbiota of high fat-fed mice is associated with increased endotoxin levels. Cani et al. have shown that administration of broad-spectrum antibiotics to mice consuming a high fat diet reduces plasma endotoxin levels to that of the control mice, while antibiotic administration to the control mice produced no significant change in plasma endotoxin (65). The presence of bifidobacteria has also been correlated with a reduction in diabetic symptoms, including improved glucose tolerance (70). Modulation of the gut microbiota may therefore be another promising strategy to prevent these prevalent metabolic issues. Furthermore, although prevention of the obese phenotype is a major clinical target, prevention of gut dysbiosis and excessive intestinal permeability will likely have other important health effects. For example, a high-fat diet can induce liver abnormalities, such as steatosis and inflammation (72), and a recent report suggests that these effects can be eliminated via regulating lipid and glucose metabolism through the consumption of BMOs and *Bifidobacterium longum* subspecies *infantis* in genetically predisposed animal models (55). Considering the interrelated nature of these and other physiological processes, the above studies may represent only a fraction of the metabolic benefits provided by milk OS.

In light of the promising effects of milk OS consumption, industrial interest in marketing these compounds for therapeutic purposes is building. As of January 2019, over 180 US patents have been filed relating to 2′-fucosyllactose alone, and the inclusion of 2′-fucosyllactose as a food ingredient is now commonplace in infant formula (73, 74). The use of 2′-fucosyllactose as an ingredient has prompted a need for large quantities of this OS to be produced synthetically. A multitude of strategies to produce synthetic OS have been designed, using both genetically engineered microorganisms and enzymatic approaches. These strategies, which have been reviewed extensively elsewhere (75), are promising routes by which individual OS could be made available at large scale. These synthetic approaches, in conjunction with the established membrane filtration strategies described above, will likely grow in application to supply the marketplace with OS that can be used as ingredients for therapeutic foods, extending availability of milk OS and their bioactivities to the general public.

## Conclusion

Growing availability of milk OS for *in vivo* experimentation is uncovering a multitude of unique and previously unknown bioactivities that could be harnessed to alleviate widespread metabolic illnesses. Future studies will likely probe further into the mechanistic details of OS functionalities, as well as evaluate the feasibility of supplementing these molecules into adult diets as therapeutics. The safety and tolerability of isolated milk OS for human consumption were recently evaluated in two independent studies and showed promising results, with even relatively high dosages being well-tolerated (76, 77). These studies could pave the way for the known metabolic impacts of BMOs to be further evaluated in human subjects, including in specialized applications such as infant formula production. Finally, implementing industrial-scale strategies to produce and isolate OS with desired bioactivities will be imperative to the application of OS as therapeutics. Therefore, we should expect continued work to identify factors influencing OS production in dairy cattle, as well as efforts to translate pilot-scale isolation techniques to dairy processing facilities.

## Author Contributions

The author confirms being the sole contributor of this work and has approved it for publication.

### Conflict of Interest Statement

The author declares that the research was conducted in the absence of any commercial or financial relationships that could be construed as a potential conflict of interest.

## Abbreviations

BMO: bovine milk oligosaccharide
Gal: galactose
Glc: glucose
GlcNAc: N-acetylglucosamine
LC-MS: liquid chromatography—mass spectrometry
OS: oligosaccharide.
